# Flexural Toughness of Steel Fiber Reinforced High Performance Concrete Containing Nano-SiO_**2**_ and Fly Ash

**DOI:** 10.1155/2014/403743

**Published:** 2014-04-23

**Authors:** Peng Zhang, Ya-Nan Zhao, Qing-Fu Li, Peng Wang, Tian-Hang Zhang

**Affiliations:** School of Water Conservancy and Environment Engineering, Zhengzhou University, No. 100 Science Road, Zhengzhou, Henan 450001, China

## Abstract

This paper aims to clarify the effect of steel fiber on the flexural toughness of the high performance concrete containing fly ash and nano-SiO_2_. The flexural toughness was evaluated by two methods, which are based on ASTM C1018 and DBV-1998, respectively. By means of three-point bending method, the flexural toughness indices, variation coefficients of bearing capacity, deformation energy, and equivalent flexural strength of the specimen were measured, respectively, and the relational curves between the vertical load and the midspan deflection (*P*_*V*_-*δ*) were obtained. The results indicate that steel fiber has great effect on the flexural toughness parameters and relational curves (*P*_*V*_-*δ*) of the three-point bending beam specimen. When the content of steel fiber increases from 0.5% to 2%, the flexural toughness parameters increase gradually and the curves are becoming plumper and plumper with the increase of steel fiber content, respectively. However these flexural toughness parameters begin to decrease and the curves become thinner and thinner after the steel fiber content exceeds 2%. It seems that the contribution of steel fiber to the improvement of flexural toughness of the high performance concrete containing fly ash and nano-SiO_2_ is well performed only when the steel fiber content is less than 2%.

## 1. Introduction

With the development of modern material science, concrete is developing in the direction of high strength, high performance, multifunction, and intelligentization. High performance concrete can be defined as the concrete which meets special performance and uniformity requirements that cannot always be achieved by conventional materials, normal mixing, placing, and curing practices [1]. Swamy states that high performance concrete is that which is designed to give optimized performance characteristics for the given set of materials, usage, and exposure conditions, consistent with requirements of cost, service life, and durability [2]. The high performance of high performance concrete includes higher strength, better workability, better volume stability, and higher durability. Architects, engineers, and constructors all over the world are finding that using high performance concrete allows them to build more durable structures at comparable cost. Particularly, high performance concrete has been widely used in high rise buildings, large span bridges, marine buildings, highways, and so on [3]. There are mainly two technical measures to obtain the high performance of high performance concrete, which are using new type high efficiency water reducing agent and mixing mineral admixture into concrete [4]. The high efficiency water reducing agent can reduce the water-binder ratio, increase the slump constant, and control the slump loss of concrete. So the concrete can achieve high density and excellent workability. Pozzolanic materials are crucial to high performance concrete as far as flowability is concerned [5]. Mineral admixtures, also called as cement replacement materials, act as pozzolanic materials as well as fine fillers, which can fill the void inside the binder, participate in the hydration reaction of the binder, increase the density of concrete, and improve the interface microstructure of concrete, so the microstructure of hardened cement matrix becomes denser and stronger and the durability and strength of concrete are improved [6].

Fly ash is the most commonly used coal combustion product and it is a remarkable material that cost effectively improves the performance of products it is added to. Its application areas include brick manufacturing and partial replacement of cement, in landfilling and reclamation of land, as soil fertilizer [7]. Being a byproduct of thermal power plants, the storage and disposal of fly ash cause considerable environmental problems [8]. Nowadays, fly ash has been used in blended cements, and it has been used successfully to replace Portland cement up to 30% by mass, without adversely affecting the strength and durability of concrete [9, 10]. The fly ash concrete offers a holistic approach that can help us to achieve the goals of meeting the rising demands for concrete, enhancement of concrete durability with little or no increase in cost (in some instances reduced cost), and ecological disposal of large quantities of the solid waste products from coal-fired power plants [9, 11]. The results of several investigations indicate that the concrete containing fly ash has exhibited excellent mechanical and durability properties [12–16].

Because nanotechnology has great market potential and economic impact, the need for research and exploration in this field and its applications has been growing significantly during the last few decades [17]. Nanotechnology has caused amazing revolution in scientific areas for the last few years. Nanoparticles are those with at least one dimension of less than 100 nanometers (nm). Nowadays, nanoparticles have been gaining growing attention and have been applied in many fields to fabricate new construction materials with novelty function owing to their unique physical and chemical properties [18]. Nanoparticles resulting from nanotechnology can act as a very active artificial pozzolan in cement-basis materials and have an influence on their structure and improve them [19]. The usage of nanoparticles in the concrete has been increasing in recent years. Among various kinds of nanomaterials, nano-SiO_2_ is the most widely used nanomaterial in the concrete. Previous study results indicated that the performance of the concrete is generally improved by adding nano-SiO_2_ [20–24]. At first, it was believed that the improvement in concrete performance due to the addition of nano-SiO_2_ is attributed to its filler effect and its pozzolanic reaction. Recently, however, it has been reported that the small particle size of nano-SiO_2_ provides a larger surface area, which speeds up the rate of cement hydration and pozzolanic reactions [17].

In the early 1900s, asbestos fibers were used in concrete, and in the 1950s the concept of composite materials came into being and fiber-reinforced concrete (FRC) was one of the topics of interest [25]. A variety of researches have been conducted to investigate the characteristics and advantages of fiber reinforced concrete in the last few decades. Fibers suitable for reinforcing concrete can be made of steel [26], glass [27], polyethylene [28], polypropylene [15], polyvinyl alcohol [29], polyester [30], aramid [31], and natural plant [32]. Among these fibers, steel fiber is one of the most popular and widely used fibers in both research and practice. Its popularity is associated with the fact that steel presents a good affinity with concrete, the ease of use, the high toughness, and resistance to static and dynamic loads [33]. Nowadays, steel fiber reinforced concrete has been successfully used in several types of construction due to the fact that steel fibers adding improves the durability and mechanical properties of hardened concrete, namely, the flexural strength, toughness, impact strength, resistance to fatigue, vulnerability to cracking, and spalling. In particular, the steel fiber reinforced concrete possesses many excellent dynamic performances such as high resistance to explosion and penetration, as compared to plain concrete and even the traditionally reinforced concrete in civil and defense engineering [34]. Owing to the favorable characteristics of steel fiber reinforced concrete, its use has steadily increased during the last two decades all over the world and its current fields of application include airport and highway pavements, earthquake-resistant and explosion-resistant structures, mine and tunnel linings, bridge deck overlays, hydraulic structures, and rock-slope stabilization [25].

Flexural toughness is extremely important for the safety and durability of structures constructed with high performance concrete, and it is so important in the mix design and application of the concrete. However, little information is presently known regarding the flexural toughness of steel fiber reinforced high performance concrete containing nano-SiO_2_ particles and fly ash. Therefore, we conducted this experimental study and measured the toughness indices (*I*_5_, *I*_10_, *I*_20_, *I*_30_), variation coefficients of bearing capacity, deformation energy, and equivalent flexure strength of the beam specimens of steel fiber reinforced high performance concrete containing nano-SiO_2_ particles and fly ash to reveal the effect of steel fibers on flexural toughness of high performance concrete containing nano-SiO_2_ and fly ash.

## 2. Experimental Program

### 2.1. Raw Materials

**Figure 1:**
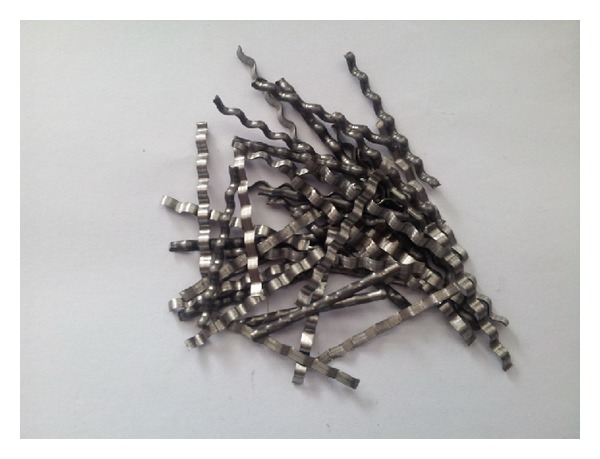
Photograph of the steel fibers used.

In this investigation, local materials were utilized. Ordinary Portland cement of Class 42.5R (which has standard compressive strength of 42.5 MPa at the age of 28 days) was used. The chemical and physical properties of the cement are presented in Table 1. Grade I fly ash was used to make the concrete for which the chemical properties are also presented in Table 1. In this experimental study, amorphous nano-SiO_2_ with a solid content of more than 99% was used. Physical properties of the nanoparticles are presented in Table 2. The steel fibers used in this study were mill-cut steel fibers for which the physical properties are presented in Table 3. A photograph of the fibers is shown in Figure 1. Coarse aggregate with a maximum size of 20 mm and fine aggregate with a 2.76 fineness modulus were used. The specific gravity and silt content of the coarse and fine aggregates were 2.74 and 0.6% and 2.63 and 0.8%, respectively. A high range water reducing agent with the name of polycarboxylate HJSX-A was used to adjust the workability of the concrete mixture. The performance indices of the high range water reducing agent are presented in Table 4. Fly ash content and nano-SiO_2_ content (by mass) are 15% and 5%, respectively, and the dosage (by volume) of steel fiber is 1.5%. The mix proportions used in this study are given in Table 5.

### 2.2. Preparation of Specimens

A series of beam specimens with the size of 100 × 100 × 400 mm were prepared to determine the flexural toughness of high performance concrete containing nano-SiO_2_ particles and fly ash. All the mixes were mixed in the mixing plant. In order to distribute nano-SiO_2_ and the steel fibers uniformly, a forced mixing machine was adopted. The mixing procedure, which was designed by trial and error, was chosen as follows: the coarse aggregate and fine aggregate were mixed initially for 1 min, and then the steel fibers were mixed for another 1 min (if required), and the cement, fly ash, and nano-SiO_2_ were mixed for another 2 min. Finally, the high range water reducer agent and water were added and mixed for 3 min. The distribution of nano-SiO_2_ and steel fibers has great effect on the working performance of the mixture and the flexural properties of the high performance concrete. If nano-SiO_2_ and steel fibers are not distributed well, they will be assembled altogether. From the working performance of the mixture and the fracture section of the specimen of the concrete, it can be seen that nano-SiO_2_ and steel fibers of this study were distributed well. After casting, all the specimens were finished with a steel towel. Immediately after finishing, the specimens were covered with plastic sheets to minimize the moisture loss from them. All the specimens were stored at temperature of about 23°C in casting room. They were demoulded after 24 h and then cured at 100% relative humidity and controlled temperature (21 ± 2°C) for 28 days before testing.

### 2.3. Flexural Toughness Test Method

**Figure 2:**
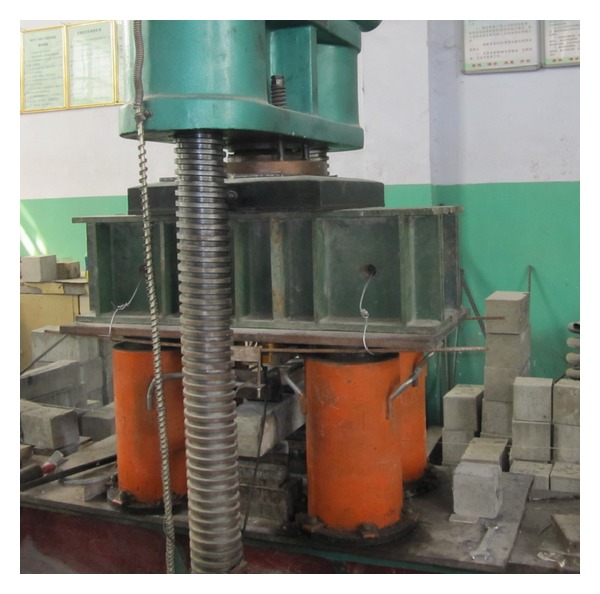
Testing apparatus of flexural toughness.

Three-point bending beam method was employed to measure the flexural toughness of steel fiber reinforced high performance concrete containing nano-SiO_2_ particles and fly ash in this study. The experiment was carried out on a hydraulic pressure testing machine with the measuring range of 2000 kN, whose measure range of the load transducer is 0–30 kN. The midspan deflection (*δ*) of the beam specimen was measured using an electrical displacement meter fixed on one side face of the specimen by an angle bracket. During the course of testing, the loading was kept continual and consistent, and the loading rate was reduced properly when the specimen was approaching failure. The loading rate of flexural toughness test was selected as 30 N/s and the average loading time was 40 min. The relational curves between the vertical load and the midspan deflection (*P*_*V*_-*δ*) were obtained, respectively, from the *X*-*Y* dynamic function recorder. The testing apparatus of flexural toughness test is presented in Figure 2.

## 3. Evaluation Methods of Flexural Toughness

### 3.1. Evaluation Method Based on ASTM C1018

**Figure 3:**
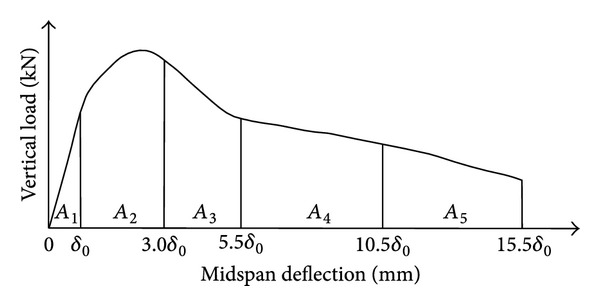
Calculation diagram for flexural toughness of ASTM method.

The evaluation method based on ASTM C1018 is a frequently used approach to determine flexural toughness of fiber reinforced concrete, which is recommended by American Society for Testing and Materials [35]. In this evaluation method, 4 flexural toughness indices (*I*_5_, *I*_10_, *I*_20_, *I*_30_) are used to evaluate the flexural toughness [36]. For the ideal elastoplastic material, the values of *I*_5_, *I*_10_, *I*_20_, and *I*_30_ are 5, 10, 15, and 30, respectively. The higher values of *I*_5_, *I*_10_, *I*_20_, and *I*_30_ indicate that the concrete has much better flexural toughness.  Figure 3 shows the typical relational curve of the vertical load and the midspan deflection (*P*_*V*_-*δ*) of flexural toughness test, and Figure 3 also illustrates the calculation of flexural toughness parameters of ASTM method. When the first crack appears, the midspan deflection of the beam specimen was defined as *δ*_0_. The flexural toughness indices *I*_5_, *I*_10_, *I*_20_, and *I*_30_ can be calculated as follows:

(1) I5 =A1+A2A1,I10 =A1+A2+A3A1,I20 =A1+A2+A3+A4A1,I30 =A1+A2+A3+A4+A5A1,

where *I*_5_, *I*_10_, *I*_20_, and *I*_30_ are flexural toughness indices; *A*_1_, the first crack area, which is the area above the axis of midspan deflection and under the curve with the midspan deflection being equal to *δ*_0_; (*A*_1_ + *A*_2_) is the area above the axis of mid-span deflection and under the curve with the midspan deflection being equal to 3*δ*_0_; (*A*_1_ + *A*_2_ + *A*_3_) is the total area above the axis of midspan deflection and under the curve with the midspan deflection being equal to 5.5*δ*_0_; (*A*_1_ + *A*_2_ + *A*_3_ + *A*_4_) is the total area above the axis of mid-span deflection and under the curve with the midspan deflection being equal to 10.5*δ*_0_; (*A*_1_ + *A*_2_ + *A*_3_ + *A*_4_ + *A*_5_) is the total area above the axis of midspan deflection and under the curve with the midspan deflection being equal to 15.5*δ*_0_.

The flexural toughness of steel fiber reinforced high performance concrete containing nano-SiO_2_ particles and fly ash also can be evaluated by the variation coefficient of bearing capacity (*ζ*_*m*,*n*_), which is corresponding to the flexural toughness index [37]. The value of *ζ*_*m*,*n*_ is closer to 1 and the concrete is closer to elastoplastic material, and the concrete has much better flexural toughness. *ζ*_*m*,*n*_ can be calculated as follows:

(2) $$\begin{array}{c} \zeta_{m,n}=\frac{I_{a\delta_{0}}-a}{a-1}, \end{array}$$

where *a*is the ratio of the given midspan deformation and critical midspan deformation, which is 3.0, 5.5, 10.5, and 15.5, respectively, in this study; *I*_*aδ*_0__ is the flexural toughness index corresponding to the given midspan deflection *aδ*_0_; *δ*_0_ is the midspan deflection of the beam specimen when the first crack appears.

### 3.2. Evaluation Method Based on DBV-1998

**Figure 4:**
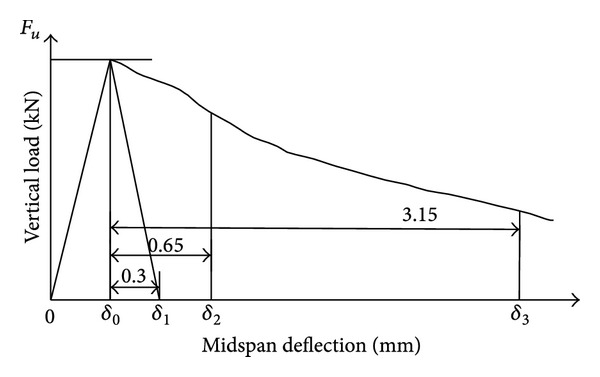
Calculation diagram for flexural toughness of DBV method.

The evaluation method based on DBV-1998 is recommended by Germany Concrete Society [38]. In this evaluation method, deformation energy and equivalent flexure strength of the beam specimens are used to evaluate the flexural toughness and the energy absorption capability of fiber reinforced.  Figure 4 illustrate the calculation of flexural toughness parameters of DBV method. The energy absorbed by the beam specimen can be calculated as follows [38]:

(3) $$\begin{array}{c} D_{n}=\overset{\delta }{\underset{0}{\int }}F\left(\delta \right)d\delta ,\\ D_{1}^{f}=D_{1}-D_{n}^{c},\\ D_{2}^{f}=D_{2}-D_{n}^{c}, \end{array}$$

where *D*_*n*_ is energy absorbed by the beam specimen (*n* = 1,2), N·m; *δ* is midspan deflection of the beam specimen, mm; *F*(*δ*) is expression of vertical load, which is connected with *δ*, kN; *D*_*n*_^*c*^ is energy absorbed by the beam specimen before cracking, which is equal to the energy absorbed when *δ* = *δ*_1_ = *δ*_0_ + 0.3 mm, N·m; *D*_1_^*f*^ is energy absorbed by the beam specimen contributed by the fibers when *δ* = *δ*_2_ = *δ*_0_ + 0.65 mm, N·m; *D*_2_^*f*^ is energy absorbed by the beam specimen contributed by the fibers when *δ* = *δ*_3_ = *δ*_0_ + 3.15 mm, N·m. The higher values of *D*_*n*_^*c*^, *D*_1_^*f*^, and *D*_2_^*f*^ indicate that the concrete has much better flexural toughness.

The flexure strength and equivalent flexure strength of steel fiber reinforced concrete containing nano-SiO_2_ particles and fly ash can be calculated as follows [38]:

(4) f =Fulbh2,Feq,1 =D1f0.5,feq,1 =Feq,1lbh2,Feq,2 =D2f3.0,feq,2 =Feq,2lbh2,

where *f* is flexural strength, MPa; *F*_*u*_ is peak vertical load, kN; *F*_eq,1_ is equivalent load when *δ* = *δ*_2_ = *δ*_0_ + 0.65 mm, kN; *F*_eq,2_ is equivalent load when *δ* = *δ*_3_ = *δ*_0_ + 3.15 mm, kN; *f*_eq,1_ is equivalent flexure strength when *δ* = *δ*_2_ = *δ*_0_ + 0.65 mm, MPa; *f*_eq,2_ is when *δ* = *δ*_3_ = *δ*_0_ + 3.15 mm, MPa; *l* is span of the beam specimen, mm; *b* is width of the beam specimen, mm; *h* is height of the beam specimen, mm. The higher values of *f*, *f*_eq,1_, and *f*_eq,2_ indicate that the concrete has much better flexural toughness.

## 4. Result and Discussion

### 4.1. Flexural Toughness Indices

**Figure 5:**
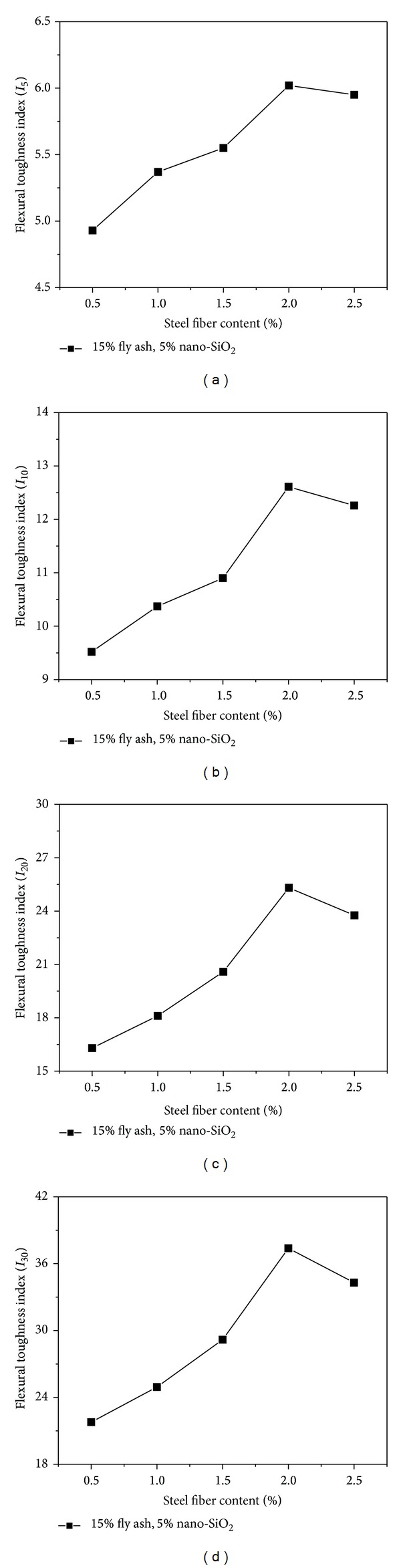
: Effect of steel fiber content on flexural toughness indices.

Figure 5 shows the variation of flexural toughness indices (*I*_5_, *I*_10_, *I*_20_, *I*_30_) of the high performance concrete containing 15% fly ash and 5% nano-SiO_2_ with the increase of steel fiber content at 28 days of curing. As can be seen from the figure, the content of steel fiber has a significant effect on flexural toughness indices. Within the steel fiber content of 2%, the flexural toughness indices are increasing gradually with the increase of steel fiber content. Compared with the concrete reinforced with 0.5% steel fiber, the increase of *I*_5_, *I*_10_, *I*_20_, and *I*_30_ was determined as 22.1%, 32.5%, 55.3%, and 71.6%, respectively, for 2% steel fiber content. However, as the steel fiber content increases continuously, great decrease in *I*_5_, *I*_10_, *I*_20_, and *I*_30_ was obtained. It seems that the addition of steel fibers has reinforcement on flexural toughness of the high performance concrete containing fly ash and SiO_2_ nanoparticles when the steel fiber content is less than 2%, and the addition of steel fibers has adverse effect of flexural toughness when the steel fiber content is beyond 2%.

From Figure 5, it can also be seen that the values of *I*_5_, *I*_10_, *I*_20_, and *I*_30_ are less than 5, 10, 20, and 30, respectively, when the steel fiber is 0.5%, which indicate that 0.5% content of steel fibers reinforced concrete containing 15% fly ash and 5% nano-SiO_2_ is definitely not an ideal elastoplastic material. Though the values of *I*_5_ and *I*_10_ are more than 5 and 10, respectively, when the steel fiber is 1%, the values of *I*_20_ and *I*_30_ are less than 20 and 30, respectively. Therefore, the concrete containing 15% fly ash and 5% nano-SiO_2_ reinforced by 1% content of steel fibers also cannot be defined as an ideal elastoplastic material. In the same way, the concrete containing 15% fly ash and 5% nano-SiO_2_ reinforced by 1.5% content of steel fibers is basically an ideal elastoplastic material, and the concrete containing 15% fly ash and 5% nano-SiO_2_ reinforced by larger content (more than 1.5%) of steel fibers is an ideal elastoplastic material. With the existence of steel fibers, the external load can be transferred to the steel fibers through the interfacial bonding between the fibers and concrete matrix. Steel fibers can restrain the crack propagation and traverse across the cracks to transfer internal force, and the steel fibers and the concrete matrix will bear the load as a whole [39]. As a result, the flexural toughness of the concrete is improved.

### 4.2. Variation Coefficient of Bearing Capacity

**Figure 6:**
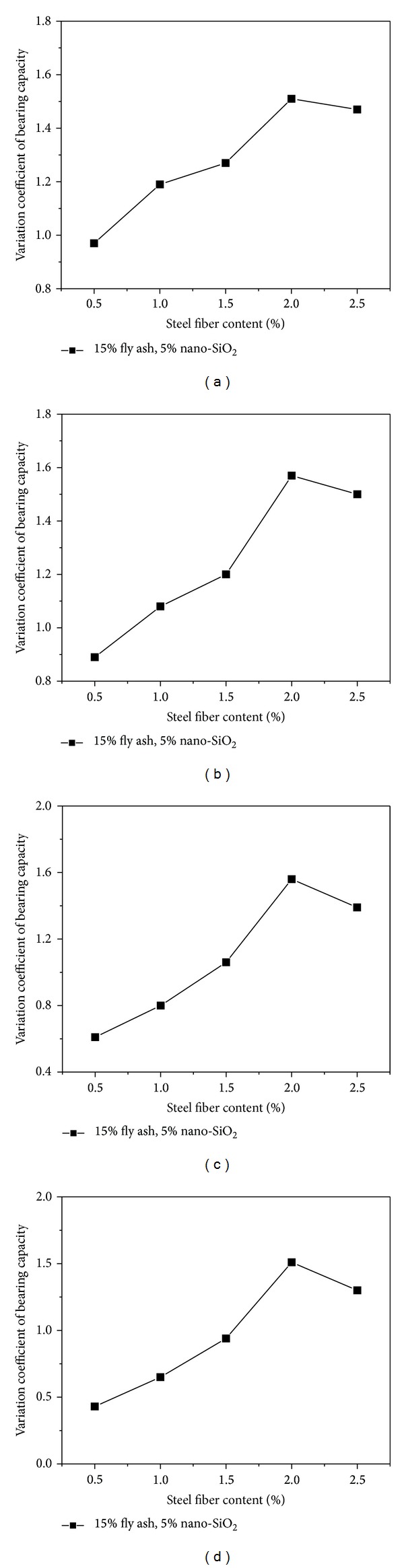
Effect of steel fiber content on variation coefficient of bearing capacity.

The varying rule of variation coefficients of bearing capacity of the high performance concrete containing 15% fly ash and 5% nano-SiO_2_ reinforced by 0.5%, 1%, 1.5%, 2%, and 2.5% dosage of steel fibers at 28 days of curing is illustrated in Figure 6. From the test results, it can be seen that the variations of variation coefficients of bearing capacity with different ratio of the given midspan deformation and critical midspan deformation (*a*) were increased with the addition of steel fibers. In other words, the concrete was transformed into elastoplastic materials from brittle materials, and they can bear much larger flexural deformation with the addition of steel fibers. When the steel fiber content is less than 2%, a considerable increase in the variation coefficient of bearing capacity with different values of *a* can be observed by increasing the content of steel fiber. When the content of steel fiber is increased from 0.5% to 2%, the variation coefficients of bearing capacity increase by 55.7% (*a* = 3.0), 76.4% (*a* = 5.5), 155.7% (*a* = 10.5), and 251.2% (*a* = 15.5), respectively. However, just like the flexural toughness indices, after the steel fiber content is beyond 2%, there is a trend of decrease in the variation coefficient of bearing capacity. With the addition of nano-SiO_2_ and fly ash, the particle size of nano-SiO_2_ and fly ash is smaller than cement, which can effectively improve the interfacial structure properties. Nano-SiO_2_ can react with Ca(OH)_2_ to form CaSiO_3_, which has filling action and improves the weak part inside the concrete. Consequently, the bonding force between the steel fibers and the concrete matrix was strengthened [40].

### 4.3. Deformation Energy

**Figure 7:**
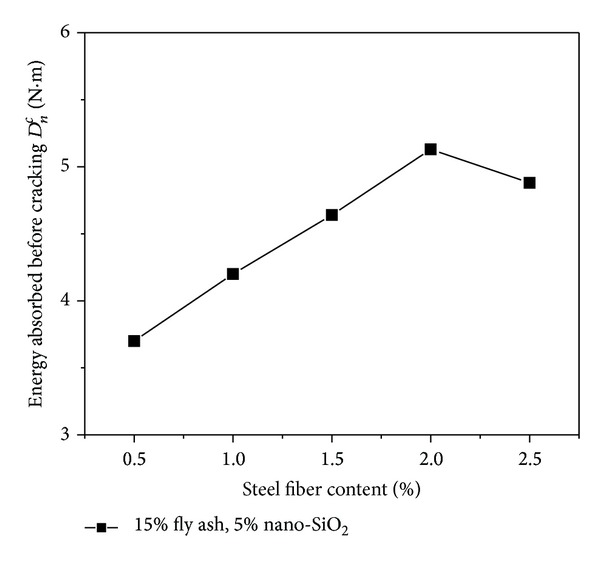
Effect of steel fiber content on energy absorbed before cracking.

Figure 7 indicates the varying rule of the energy absorbed by the beam specimen before cracking (*D*_*n*_^*c*^) for the high performance concrete (15% fly ash and 5% nano-SiO_2_) reinforced by different dosage of steel fibers. From the figure, it can be seen that the addition of steel fibers can increase the energy absorbed by the beam specimen before cracking effectively. As can be seen from Figure 7, the variation rule of *D*_*n*_^*c*^ of the concrete containing fly ash and nanoparticles with the increase of steel fiber content is similar to that of flexural toughness indices. That is, the value of *D*_*n*_^*c*^ is increased gradually with the increase of steel fiber content and *D*_*n*_^*c*^ reaches a maximum when the steel fiber content is 2%, while *D*_*n*_^*c*^ drops down after the steel fiber content is beyond 2%.

**Figure 8:**
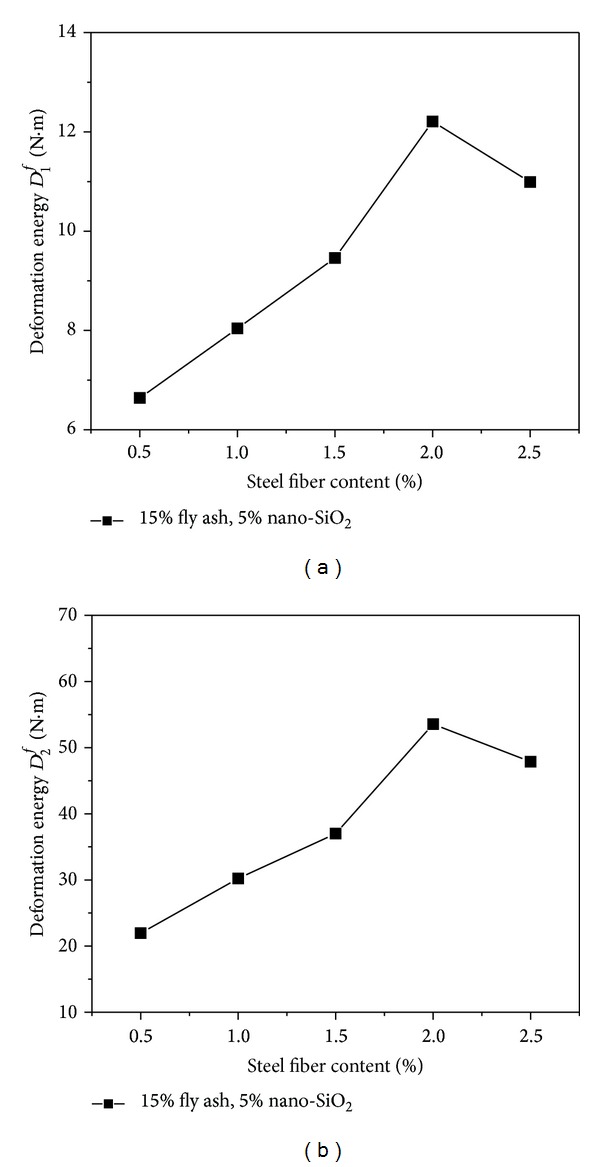
Effect of steel fiber content on deformation energy.

Figure 8 presents the varying rules of the deformation energy (*D*_1_^*f*^ and *D*_2_^*f*^) of the high performance concrete containing 15% fly ash and 5% nano-SiO_2_ with the increase of steel fiber content. Both of the deformation energies (*D*_1_^*f*^ and *D*_2_^*f*^) were increased by adding steel fibers into the concrete. As the dosage of steel fiber is increasing from 0.5% to 2%, either *D*_1_^*f*^ or *D*_2_^*f*^ has a trend of increase, and they have peak values when the steel fiber content reaches 2%. The increases of the *D*_1_^*f*^ and *D*_2_^*f*^ were determined as 65.5% and 118.1% for the concrete with 2% steel fiber content, respectively. As the content of steel fiber exceeds 2%, both of the values of *D*_1_^*f*^ and *D*_2_^*f*^ begin to decrease slightly. After the concrete matrix cracks, because of the preferable bonding strength between the steel fiber and the concrete matrix, the steel fibers have strong deformability. During the course of the steel fibers being pulled out or being pulled off, the fibers absorb large quantities of energy. As a result, the flexural toughness of the concrete containing fly ash and nano-SiO_2_ was improved by the steel fibers.

### 4.4. Equivalent Flexure Strength

**Figure 9:**
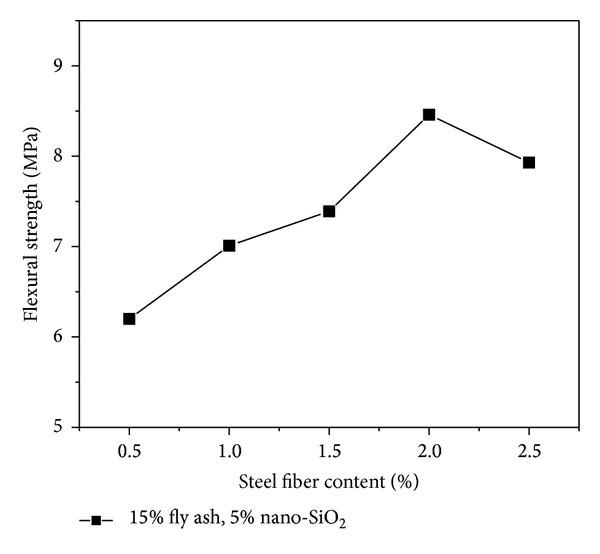
Effect of steel fiber content on flexural strength.

**Figure 10:**
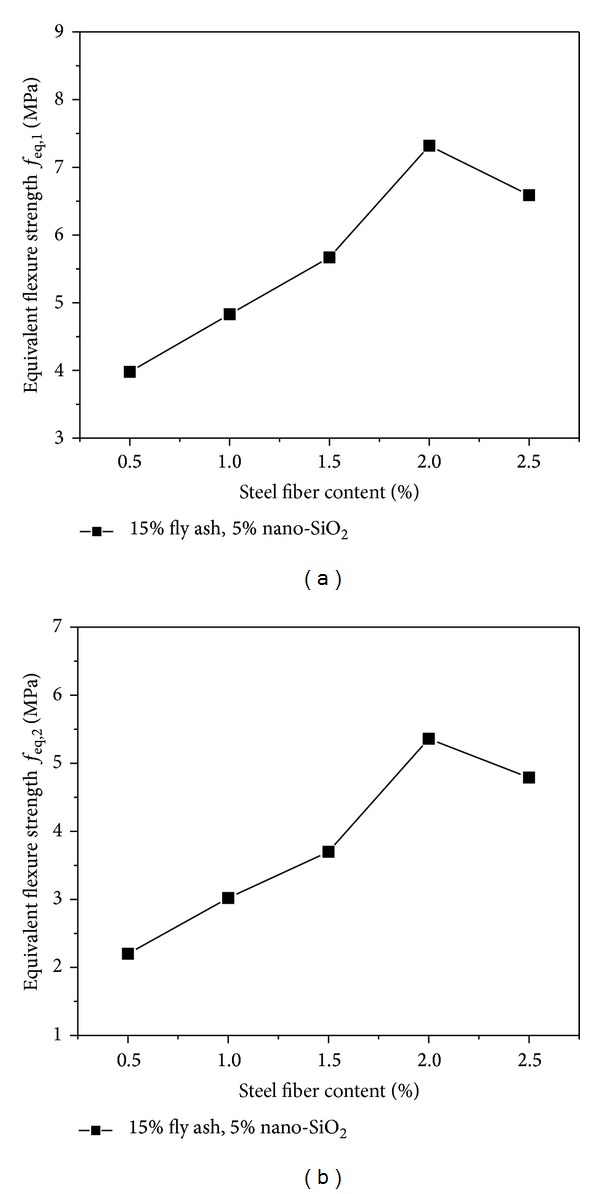
Effect of steel fiber content on equivalent flexure strength.

The relation curve between the flexural strength and steel fiber content of the high performance concrete containing 15% fly ash and 5% nano-SiO_2_ at 28 days of curing period is given in Figure 9. Increasing steel fiber content, a considerable increase in the flexural strength of the mixes was obtained; for example, compared with the concrete reinforced with 0.5% steel fibers, the increases were determined as 36.5% for steel fiber content of 2% at 28 days of curing period. After the flexural strength reaches maximum value with the steel fiber content of 2%, the value of flexural strength begins to drop down. As is shown in Figure 10, steel fiber content also has great effect on the equivalent strengths *f*_eq,1_ and *f*_eq,2_. Similar with the flexural strength *f*, *f*_eq,1_ and *f*_eq,2_ are increasing gradually with the increase of steel fiber content when the steel fiber content is less than 2%, while they begin to decrease when the fiber content exceeds 2%.

**Figure 11:**
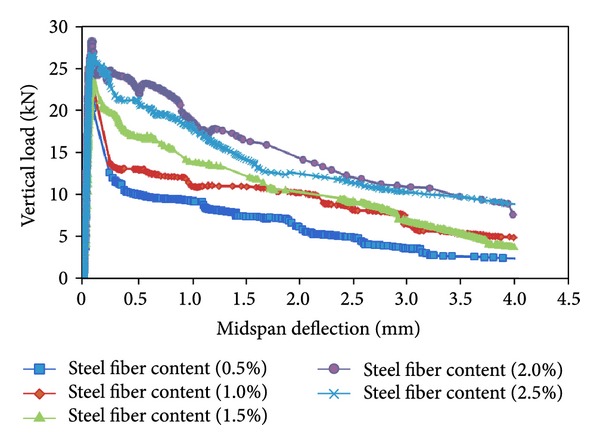
Contrast of “*P*_*V*_-*δ*” curves of different steel fiber content.

Figure 11 presents the typical complete curves of *P*_*V*_-*δ* of the bending beam specimens of the high performance concrete containing 15% fly ash and 5% nano-SiO_2_ with different steel fiber contents. From Figure 11, it can be seen that the descent parts of the curves of the specimen with lower steel fiber content (0.5% and 1.0%) are very steep. In other words, the peak vertical load sharply decreased with very little increase in midspan deflection of the beam specimen. However, the descent parts of the curves of the specimen with higher steel fiber content (1.5%, 2%, and 2.5%) are very smooth and gentle. Besides, the relational curve of *P*_*V*_-*δ* becomes plumper and plumper when the steel fiber content increases from 0.5% to 2%, while the relational curve of *P*_*V*_-*δ* becomes thinner and thinner when the steel fiber exceeds 2%. For the concrete, the plumper relational curve of *P*_*V*_-*δ* indicates that the bending beam specimen has better flexural toughness. Accordingly, if the steel fiber content is limited in a certain range, the steel fibers have great improvement on the flexural toughness of the concrete containing fly ash and SiO_2_ nanoparticles.

The steel fibers play an important role in bridging microcracks in the high performance concrete containing fly ash and nanoparticles, and the cohesive force between the concrete matrix and the steel fibers with high elasticity modulus has a certain anticracking effect. Besides, with the addition of steel fibers in the concrete, the disordered fibers distributing inside the concrete in three dimensions can form a structural support inside the concrete, and the strength of the concrete matrix can be improved. Under the loads, inside the concrete, some microcracks will appear and there will be high stress concentration on the crack tip. When the crack tip reaches the steel fibers, because the strength of steel fiber is much higher than that of the concrete matrix, and the size of the steel fiber is larger than the crack tip, the steel fibers can prevent the propagation of the cracks. As a result, with the appropriate fiber content, the addition of steel fibers can improve the flexural properties of the concrete containing fly ash and nanoparticles. However, if the steel fiber content is too high, the large amount of fibers may increase the number of microcracks and cause some defects inside the concrete. Therefore, the steel fiber content of more than 2% will decrease *f*, *f*_eq,1_, and *f*_eq,2_ of the concrete containing fly ash and nano-SiO_2_.

## 5. Conclusions

This paper reported experimental results of flexural toughness studies conducted on fly ash and nano-SiO_2_ high performance concrete reinforced with steel fibers. The following conclusions can be draw from the results presented in this paper.

1. Addition of steel fibers has great improvement on the flexural toughness indices (*I*_5_, *I*_10_, *I*_20_, *I*_30_) and variation coefficients of bearing capacity (*ζ*_*m*,*n*_) of the concrete containing 15% fly ash and 5% nano-SiO_2_. The values of *I*_5_, *I*_10_, *I*_20_, *I*_30_, and *ζ*_*m*,*n*_ are increased gradually with the increase of steel fiber content and they reach the maximum when the steel fiber content is 2%, while these parameters drop down after the steel fiber content is beyond 2%. These variation rules of flexural toughness parameters indicate that the contribution of steel fibers to the flexural toughness of the concrete containing fly ash and nano-SiO_2_ is well performed only when the steel fiber content does not exceed 2%.
2. Effect of steel fibers on the deformation energy (*D*_*n*_^*c*^, *D*_1_^*f*^, *D*_2_^*f*^) and (equivalent) flexural strength (*f*, *f*_eq,1_, *f*_eq,2_) of the high performance concrete containing fly ash and nano-SiO_2_ is significant. Besides, steel fiber has great effect on the relational curves of *P*_*V*_-*δ* of the bending beam specimen. When the content of steel fiber increases from 0.5% to 2%, *D*_*n*_^*c*^, *D*_1_^*f*^, *D*_2_^*f*^, *f*, *f*_eq,1_, and *f*_eq,2_ increase gradually and the relational curves of *P*_*V*_-*δ* are becoming plumper and plumper. However these parameters begin to decrease and the curves become thinner and thinner after the steel fiber content exceeds 2%. The variation rules of these parameters and relational curves indicate that the capability of steel fiber to resist crack propagation of the concrete containing fly ash and nano-SiO_2_ is becoming stronger and stronger with the increase of fiber content with the fiber content not beyond 0.12%.

## Figures and Tables

**Table 1:** Properties of cement and fly ash.

Composition (%)	Cement	Fly ash
Chemical compositions		
SiO_2_	20.85	51.50
Al_2_O_3_	5.32	18.46
Fe_2_O_3_	2.69	6.71
CaO	62.97	8.58
MgO	3.66	3.93
Na_2_O	0.15	2.52
K_2_O	0.62	1.85
SO_3_	2.48	0.21
Physical properties		
Specific gravity	3.11	2.16
Specific surface (cm^2^/g)	3287	2470

**Table 2:** Physical properties of nano-SiO_2_.

Average particle size (nm)	SiO_2_ content (%)	Specific surface area (m^2^/g)	Apparent density (g/cm^3^)	pH value
30	99.5	200 ± 10	0.055	5–7

**Table 3:** Properties of steel fibers.

Length (mm)	Equivalent diameter (mm)	Length diameter ratio	Tensile strength (MPa)
32	0.56	52.0	800

**Table 4:** Properties of high range water reducing agent.

Solid content (%)	Total alkali content (%)	Fluidity of cement paste (mm)	Density (g/cm^3^)	Content of Cl^−^ (%)	pH value
30	1.2	260	1.052	0.078	4.32

**Table 5:** Mix proportions of the concrete composites.

Mix number	Cement (kg/m^3^)	Fly ash (%)	Nano-SiO_2_ (%)	Steel fiber (%)	Fine aggregate (kg/m^3^)	Coarse aggregate (kg/m^3^)	Water (kg/m^3^)	Water reducing agent (kg/m^3^)
1	395.2	15	5	0.5	647	1151	158	4.94
2	395.2	15	5	1	647	1151	158	4.94
3	395.2	15	5	1.5	647	1151	158	4.94
4	395.2	15	5	2	647	1151	158	4.94
5	395.2	15	5	2.5	647	1151	158	4.94
